# Effects of chemerin and homocysteine levels and their associations with occurrence and development of ischemic cerebrovascular disease

**DOI:** 10.1186/s12944-021-01524-7

**Published:** 2021-09-20

**Authors:** Lixuan Wang, Jianpu Jia, Zhen Hong, Leguo Zhang, Junling Zhang

**Affiliations:** Department of Neurology, Cangzhou Central Hospital, No.16 West Xinhua Road, Hebei 061000 Cangzhou, China

**Keywords:** Ischemic cerebrovascular disease, Serum homocysteine, Serum adipocytokine, Occurrence and development, Correlation

## Abstract

**Background:**

The current study was conducted to explore the effects of chemerin and homocysteine (Hcy) levels and their associations with the occurrence and development of ischemic cerebrovascular disease (ICVD).

**Methods:**

There involved a total of 187 patients with ICVD and 190 healthy people for physical examination in Cangzhou Central hospital from January 2020 to April 2021. The participants enrolled were divided into four groups based on the digital subtraction angiography: mild stenosis group (64 cases, stenosis rate 30-49 %), moderate stenosis group (72 cases, stenosis rate 50-69 %), severe stenosis group (51 cases, stenosis rate 70-99 %) and control group (190 cases, in healthy condition). The laboratory indexes of ICVD group and control group were observed and the four groups were further compared. Pearson linear correlation was applied to analyze the link between chemerin and Hcy levels and the degree of cerebral vascular stenosis in ICVD patients, and multivariate logistic regression was used to analyze the influencing factors of ICVD.

**Results:**

No significant difference was found in general information including age, gender, body mass index (BMI), heart rate, systolic blood pressure, diastolic blood pressure, smoking and drinking between the two groups (*P* > 0.05). Moreover, there was no significant difference in fasting blood glucose (FBG), total cholesterol (TC) and high density lipoprotein cholesterol (HDL-C) levels between the two groups (*P* > 0.05). However, the levels of triglyceride (TG), low density lipoprotein cholesterol (LDL-C), chemerin and Hcy in ICVD group were significantly higher than those in control group (*P* < 0.05). When comparing the four groups, there was no significant difference in FBG and TC levels (*P* > 0.05). The levels of TG, LDL-C, chemerin and Hcy in mild, moderate and severe stenosis groups were higher than those in control group, the above levels in moderate and severe stenosis group were higher than those in mild stenosis group, and severe stenosis group higher than moderate stenosis group (*P* < 0.05). Chemerin and Hcy levels were positively correlated with the degree of cerebral vascular stenosis in ICVD patients (*r* = 0.612, 0.519, *P* < 0.001). ICVD was regarded as the dependent variable, and the abovementioned general data as well as significant laboratory indicators, including TG, LDL-C, chemerin and Hcy, as independent variables. The results of multivariate logistic regression analysis revealed that TG, LDL-C, chemerin and Hcy were independent influencing factors of ICVD.

**Conclusions:**

Chemerin and Hcy levels exerted a close link to the occurrence and development of ICVD as independent influencing factors.

## Introduction

Ischemic cerebrovascular disease (ICVD), known as a common clinical ischemic disease, including lacunar infarction, transient ischemic attack and progressive stroke, accounts for about 70 % of all stroke [1]. As China’s aging population growing and changes of eating habits, the ischemic cerebrovascular diseases have experienced a trend towards a gradual increase in recent years, which brings a negative impact on patients, their families and society. Nowadays this condition is no doubt a tricky issue for public health [2]. Regarding the pathological basis of the disease, that ischemic necrosis of local brain tissue occurs following atherosclerosis, digital subtraction angiography has been considered as the gold standard for clinical diagnosis of ischemic cerebrovascular disease [3]. The technology is able to clearly show the degree of intracranial and extracranial artery stenosis and provide reliable reference for disease assessment [4]. But it is an invasive examination, so limitation exists for repetitive operation and clinical promotion. Serum adipocytokine (chemerin) is a secreted protein and an important metabolic factor in the body. It can induce different progress of glycolipid metabolism and inflammatory response through the secretion status of adipose tissue bank, including the phenotype and location of immune cells, structural cells and the vascular cells. Hence earlier studies found its link to obesity, metabolic syndrome and atherosclerosis [5]. Serum homocysteine (Hcy) is highly expressed in patients with cardiovascular and cerebrovascular diseases. Relevant studies have confirmed that Hcy is closely related to stroke [6]. However, there are limited clinical reports on the effects of chemerin and Hcy levels on the occurrence and development of ICVD. Herein, the present study sought to investigate the potential correlation between chemerin and Hcy levels and the occurrence and development of ICVD.

## Materials and methods

### Participants

There involved a total of 187 patients harboring ICVD (ICVD group) and 190 healthy people for physical examination (control group) between January 2020 to April 2021. The patients were divided into mild stenosis group (64 cases, stenosis rate 30-49 %), moderate stenosis group (72 cases, stenosis rate 50-69 %), severe stenosis group (51 cases, stenosis rate 70-99 %) based on the digital subtraction angiography. In ICVD group, there were 101 males and 86 females, aged 47 to 69 years, average (51.98 ± 9.78) years of age. In control group: there were 107 males and 83 females, aged 48–70 years old, average (51.91 ± 9.79) years of age. The two groups were comparable in age and gender (*P* > 0.05). The current research program meets the requirements of the declaration of Helsinki of the World Medical Association.

### Selection criteria

#### Inclusion criteria

Patients enrolled were diagnosed via computed tomography (CT) or magnetic resonance imaging (MRI), in accordance with the criteria of Chinese guidelines for the prevention and treatment of cerebrovascular diseases, and cerebral artery stenosis was found by digital subtraction; the patients were diagnosed after the first attack; informed consent was obtained and signed; no abnormal coagulation function was found; patients had no problem in mental health. Healthy people for physical examination were confirmed through a comprehensive physical examination, laboratory examination, and imaging examination including CT and MRI.

#### Exclusion criteria

Patients who met the following criteria were excluded: stenosis rate < 30 %; complicated with severe liver and kidney dysfunction (elevation of transaminase, urea nitrogen and serum creatinine, etc.) ; complicated with cardiac diseases (heart failure, hypertensive heart disease, myocardial infarction, myocarditis, etc.); complicated with arteritis, blood disease, tumor, cerebrovascular malformation or aneurysm; complicated with post-infarction hemorrhage or hemorrhagic stroke; complicated with autoimmune diseases and endocrine system (systemic lupus erythematosus, rheumatoid arthritis, hyperthyroidism, etc.); taking lipid-lowering drugs; history of surgical operation or tissue trauma, acute or chronic infection or chronic inflammatory disease within the past 3 months; recent use of folic acid, B vitamins or methotrexate; complicated with thyroid disease.

## Methods

### Digital subtraction angiography and grouping

ICVD patients were examined by digital subtraction angiography. The same group of radiologists completed the examination of intracranial and extracranial artery. The intracranial artery included intracranial segment of internal carotid artery, M1 segment of middle cerebral artery, intracranial segment of vertebral artery and basilar artery. The extracranial artery included common carotid artery, internal carotid artery, extracranial segment of vertebral artery and subclavian artery.

According to the standard of the North American Symptomatic Carotid Endarterectomy Trial (NASCET) [7], the diameter of the narrowest part of the cross-section of the vessel (N) and the diameter of the distal normal vessel (D) were measured, and then the vascular stenosis rate was calculated by using the formula: vascular stenosis rate = (1-N/D) ×100 %. On the basis of the degree of stenosis, patients with 30-49 % stenosis were classified as mild stenosis group, 50-69 % as moderate stenosis group, 70-99 % as severe stenosis group—total occlusion. Stenosis rate less than 30 % was excluded due to the difficulty for the selection of vessels. Two experienced neurointerventional physicians performed the digital subtraction angiography and the assessment of vascular stenosis. The extracranial artery (common carotid artery, subclavian artery, extracranial segment of internal carotid artery and extracranial segment of vertebral artery) and intracranial artery (intracranial segment of internal carotid artery, M1 segment of middle cerebral artery, intracranial segment of vertebral artery and basilar artery) were evaluated. If the patient has stenosis in multiple parts of an artery, the one with the most severe stenosis will be selected. In addition, healthy people were assigned as the control group.

### Background collection and physical examination

The self-made questionnaires were applied by two trained medical staff, including: age, gender, body mass index (BMI), smoking, history of hypertension, coronary artery disease, diabetes, dyslipidemia, etc. Height, weight, heart rate and blood pressure were measured.

### Laboratory examination

Three-five milliliters of fasting elbow venous blood was collected from all participants and stored in -40 ℃ refrigerator after centrifugation. Roche 7600 automatic biochemical analyzer (Swiss) was used to detect the biochemical indexes of patients, including fasting blood glucose (FBG), total cholesterol (TC), triglyceride (TG), low density lipoprotein cholesterol (LDL-C), high density lipoprotein cholesterol (HDL-C), etc. Chemerin level was detected by the double-antibody sandwich enzyme-linked immunosorbent assay (Runyu Biotechnology Co., Ltd, Shanghai). Hcy level was detected by microparticle enzyme-linked immunosorbent assay (Jinma Biotechnology Co., Ltd, Shanghai).

### Observation indexes

The laboratory indexes of ICVD group and control group were observed and four groups were compared. Pearson linear correlation was applied to analyze the correlation between chemerin and Hcy levels and the degree of cerebral vascular stenosis in ICVD patients, and multivariate logistic regression was used to analyze the influencing factors of ICVD.

### Statistical analysis

All analyses were undertaken using SPSS 23.0 version. Measurement data conforming to normal distribution are expressed by mean ± SD and the comparison between the groups was analyzed by t-test. Counting data were compared by chi-square test. Spearman correlation analysis was used to analyze the correlation between Chemerin and Hcy levels and the degree of cerebrovascular stenosis in patients with ICVD; the influencing factors of ICVD were analyzed by Logistic regression. All tests were two-tailed. *P* < 0.05 was considered significant for all statistical analysis.

## Results

### Comparison of general data between the two groups

There was no significant difference in age, gender, BMI, heart rate, systolic blood pressure, diastolic blood pressure, tobacco use and drinking between the two groups (*P* > 0.05), as shown in Table 1.

**Table 1:** Comparison of general data between the two groups

Item	ICVD group (187)	Control group (190)	t/χ^2^	*P*
Age (year)	51.98 ± 9.78	51.91 ± 9.82	0.009	0.992
Sex (male/female)	101/86	107/83	0.203	0.653
BMI (kg/m^2^)	23.26 ± 1.69	23.03 ± 1.53	1.386	0.167
Heart rate (/min)	72.87 ± 9.93	71.92 ± 10.06	0.923	0.357
Systolic blood pressure (mmHg)	116.29 ± 11.65	114.32 ± 11.72	1.645	0.101
Diastolic blood pressure (mmHg)	75.63 ± 8.69	76.02 ± 8.73	-0.435	0.664
Combined diseases (n)				
Hypertension	88	0	116.637	< 0.001
Diabetes	26	0	28.734	< 0.001
Coronary artery disease	10	0	10.788	0.001
Dyslipidemia	62	0	75.394	< 0.001
Tobacco use	60	51	0.086	0.769
Alcohol drinking	62	58	0.299	0.584

*BMI* body mass index

### Comparison of laboratory indexes between the two groups

There was no significant difference in FBG, TC and HDL-C levels between the two groups (*P* > 0.05). The levels of TG, LDL-C, chemerin and Hcy in ICVD group were significantly higher than those in control group (*P* < 0.05), as laid out in Table 2.

**Table 2:** Comparison of laboratory indexes between the two groups

Item	ICVD group (187)	Control group (190)	t	*P*
FBG(mmol/L)	5.76 ± 1.02	5.59 ± 1.01	1.626	0.105
TC(mmol/L)	4.81 ± 1.06	4.65 ± 1.09	1.445	0.149
TG(mmol/L)	1.91 ± 0.31	1.21 ± 0.28	23.015	< 0.001
LDL-C(mmol/L)	3.46 ± 0.53	2.34 ± 0.51	20.309	< 0.001
HDL-C(mmol/L)	1.22 ± 0.37	1.29 ± 0.39	-1.787	0.075
Chemerin(mg/mL)	104.39 ± 7.78	77.31 ± 7.69	32.733	< 0.001
Hcy(µmol/L)	10.32 ± 1.82	6.29 ± 1.76	21.856	< 0.001

*FBG* fasting blood glucose, *TC* total cholesterol, *TG* triglyceride, *LDL-C* low density lipoprotein cholesterol, *HDL-C* high density lipoprotein cholesterol, *Hcy* homocysteine

### Comparison of laboratory indexes in each group

No significant difference was observed in FBG, TC and HDL-C levels among the four groups (*P* > 0.05). The levels of TG, LDL-C, chemerin and Hcy in mild, moderate and severe stenosis groups were higher than those in control group, the above levels in moderate and severe stenosis group were higher than those in mild stenosis group, and severe stenosis group higher than moderate stenosis group (*P* < 0.05), as laid out in Table 3.

**Table 3:** Comparison of laboratory indexes in each group

Item	Mild stenosis group (64)	Moderate stenosis group (72)	Severe stenosis group (51)	Control group (190)	t	*P*	
FBG(mmol/L)	5.32 ± 1.23	5.39 ± 1.12	5.48 ± 1.03	5.59 ± 1.01	1.293	0.277	
TC(mmol/L)	4.61 ± 1.27	4.73 ± 1.13	4.79 ± 1.22	4.65 ± 1.09	0.324	0.808	
TG(mmol/L)	1.91 ± 0.32^a^	2.18 ± 0.31^ab^	2.34 ± 0.32^abc^	1.21 ± 0.28	320.111	< 0.001	
LDL-C(mmol/L)	2.87 ± 0.59^a^	3.58 ± 0.53^ab^	4.11 ± 0.55^abc^	2.34 ± 0.51	196.282	< 0.001	
HDL-C(mmol/L)	1.38 ± 0.35	1.36 ± 0.34	1.31 ± 0.33	1.29 ± 0.39	1.305	0.272	
Chemerin(mg/mL)	89.92 ± 7.63^a^	101.29 ± 7.69^ab^	115.87 ± 7.92^abc^	77.31 ± 7.69	413.108	< 0.001	
Hcy(µmol/L)	8.07 ± 1.81^a^	9.52 ± 1.83^ab^	11.93 ± 1.91^abc^	6.29 ± 1.76	154.864	< 0.001	

*FBG* fasting blood glucose, *TC* total cholesterol, *TG* triglyceride, *LDL-C* low density lipoprotein cholesterol, *HDL-C* high density lipoprotein cholesterol, *Hcy* homocysteine

^a^compared with the control group, *P* < 0.05

^b^compared with mild stenosis group, *P* < 0.05

^c^compared with moderate stenosis group, *P* < 0.05

### Correlation analysis of chemerin and Hcy levels with the degree of cerebral vascular stenosis in patients with ICVD

Chemerin and Hcy levels were positively correlated with the degree of cerebral vascular stenosis in ICVD patients (*r* = 0.612, 0.519, *P* < 0.001).

### The influencing factors of ICVD by multivariate logistic regression analysis

ICVD was regarded as the dependent variable, and the abovementioned general data and laboratory indicators, including TG, LDL-C, chemerin and Hcy, as independent variables. Multivariate logistic regression analysis showed that TG, LDL-C, chemerin and Hcy were independent influencing factors of ICVD, as shown in Table 4.

**Table 4:** Multivariate logistic regression analysis on influencing factors of ICVD

Influencing factor	β value	Wald χ^2^ value	*P* value	OR value (95 %CI)
TG	0.323	6.061	0.016	3.698(2.401–4.187)
LDL-C	0.031	7.193	0.011	4.198(3.103–6.212)
Chemerin	0.231	5.798	0.017	1.368(1.103–1.832)
Hcy	0.476	10.239	0.001	1.143(0.946–1.494)

*TG* triglyceride, *LDL-C* low density lipoprotein cholesterol, *Hcy* homocysteine

## Discussion

Statistics show that the incidence rate of ICVD has experienced a trend with a gradual increase year by year with younger patients. The condition causes negative impact on the physical and mental health of the patients and brings heavy financial burden to their families as well as society. Therefore, early diagnosis and targeted intervention remain pivotal to clinical practice [8].

Currently, the pathogenesis of ICVD has not been fully understood, but atherosclerosis has been widely recognized as the main pathological change. Lots of focus has been laid on adipokines and atherosclerosis in recent years [9]. Current studies believe that atherosclerosis is a chronic inflammatory reaction process, and circulating pro-inflammatory adipokines are a chronic inflammatory reaction process. The imbalance of circulating pro-inflammatory adipokines and anti-inflammatory adipokines jointly promote the development of atherosclerosis and related diseases [10]. On one hand, chemerin is considered to be a chemotactic protein involved in early inflammatory response. It can act as pro-inflammatory role, recruit monocyte derived macrophages expressing chemerin receptor (chemr23) to the site of inflammatory injury, activate chemotactic macrophages and other immunocompetent cells through chem/chemr23 interaction to release tumor necrosis factor-α (TNF- α), interleukin-6 (IL-6) and other inflammatory factors [11]. Moreover, several studies are available to show that chemerin can promote accumulation of macrophage cholesterol and transformation of macrophage foam cell for atherosclerosis formation [12]. According to Demir et al., chemerin was associated with ischemic cerebrovascular disease [13]. On the other hand, Hcy is an important intermediate metabolite of sulfur-containing amino acids in the body. High level of Hcy can lead to the formation of atherosclerotic plaque, platelet aggregation and adhesion. It can also damage vascular endothelial cells, vascular elastic layer, and the function of blood flow regulation, which has negative impact on vascular regeneration and collateral circulation. Hcy is therefore considered as an independent risk factor for atherosclerosis and stroke, which can aggravate the condition of patients with ischemic stroke [14]. Yu et al. [15] demonstrated that the blood coagulation of ICVD patients is in hypercoagulable state, and the combination of Hcy and TEG detection could provide the basis for clinical treatment, efficacy and prognosis evaluation. The results of this present study showed that the levels of TG, LDL-C, chemerin and Hcy in ICVD group were higher than those in control group (*P* < 0.05), and the levels of TG, LDL-C, chemerin and Hcy in mild, moderate, and severe stenosis groups were higher than those in control group. The levels of TG, LDL-C, HDL-C, chemerin and Hcy in mild stenosis group were lower than those in moderate and severe stenosis groups, and HDL-C in moderate and severe stenosis groups were higher than that in control group (*P* < 0.05). It was suggested that the levels of TG, LDL-C, chemerin, Hcy and HDL-C were high in patients with ICVD, and serious stenosis degree was positively linked to high level of indexes.

According to the study supported by Zhao et al. [16], the expression of serum chemerin was up-regulated in patients with ischemic stroke. Previous study found that the level of chemerin factor was related to blood pressure, lipid and inflammatory reaction in patients with ICVD, and the increase of chemerin factor level was accompanied by the aggravation of arterial stenosis and the progress of ICVD, which was expected to become a reference index for clinical evaluation and prognosis of ICVD [17]. Yao et al. [18] documented that the degree of carotid artery stenosis and the feature of plaque in ICVD patients were linked to serum Hcy. High level of Hcy was able to increase the incidence of carotid artery stenosis and unstable plaque, further aggravating the patient’s condition. According to Fu et al. [19], cyst-c, matrix metalloproteinase-9 (MMP-9) and Hcy as independent risk factors for cap formation were significantly increased with the increase of the degree of carotid atherosclerosis in ICVD patients. Previous studies showed that the level of Hcy in patients with ischemic stroke was significantly higher than that in the control group, suggesting that the level of Hcy might be a risk factor for ischemic stroke, and that Hcy improved the predictive ability of traditional risk factors for stroke [20, 21]. The results of this study showed that the levels of chemerin and Hcy in ICVD patients were positively correlated with the degree of cerebral vascular stenosis (*P* < 0.001), suggesting that the occurrence and development of ICVD were highly associated with the levels of chemerin and Hcy. Regarding the occurrence and development of ICVD are closely linked to atherosclerosis, the possible mechanisms of chemerin and ICVD are summarized as follows: (1) chemerin can promote the production of adipocytes, resulting in lipid metabolism disorder and insulin resistance; (2) IL-6, tumor necrosis factor-α (TNF-α) could up-regulate the expression of chemerin receptor in vascular endothelial cells; (3) chemerin factor has the ability to induce monocytes, which can up-regulate the expression of monocyte adhesion molecules and promote monocyte aggregation under the vascular endothelium. These mechanisms could lead to abnormal lipid metabolism, the occurrence and development of inflammatory reaction, and promote atherosclerosis, resulting in artery stenosis and unstable plaque progression, thus inducing ICVD. The possible mechanisms of Hcy and ICVD are as follows: (1) Hcy is able to damage vascular endothelium; (2) Hcy can stimulate the abnormal proliferation of vascular smooth muscle cells; (3) Hcy can cause platelet aggregation, change blood viscosity and increase blood coagulation; (4) Hcy is able to enhance the peroxidation of LDL-C, increase the production of LDL-C, accelerate the progression of atherosclerosis, hence aggravate the condition of ICVD [22]. Kang et al. [23] showed that LDL-C and transcranial Doppler (TCD) detection can predict the occurrence of ischemic cerebrovascular disease, which has high application value for early diagnosis and intervention of ICVD. Liu et al. [24] documented that the serum chemerin expression level was increased in patients with acute cerebral infarction, which can be used as a biological diagnostic marker for acute cerebral infarction. Pang et al. [25] also pointed out that the increase of Hcy level was related to the incidence of ischemic stroke, affecting the cerebrovascular health. And it was positively correlated with the degree of stroke, which can be used to evaluate and predict the prognosis of patients. The current study confirmed that TG, LDL-C, chemerin and Hcy were independent influencing factors of ICVD.

### Comparison with other studies

At present, the gold standard in clinical diagnosis of ischemic cerebrovascular disease is digital subtraction angiography. Digital subtraction angiography is able to clearly show the degree of intracranial and extracranial artery stenosis and provide reliable reference for disease assessment [4]. However, it is an invasive examination, which exists limitation for repetitive operation and clinical promotion. The advantage of this study is that a relatively non-invasive detection method is proposed. The results of present study confirmed that Chemerin and Hcy levels were closely related to the occurrence and development of ICVD, and were independent factors affecting ICVD.

### Study strength

Few studies reported the relationship between ischemic cerebrovascular disease and Hcy. This study revealed that chemerin and Hcy levels exerted a close link to the occurrence and development of ICVD as independent influencing factors. Hence, the results of this study provided a promising indicator for clinical monitoring of ischemic cerebrovascular disease.

### Limitations

This study still existed several limitations. Firstly, this study was a retrospective study based on the previous inpatients, bias exist for enrollment. Additionally, there involved a few risk factors of ischemic cerebrovascular disease, further accurate and prospective researches with larger sample size are warranted on the characteristics of cerebral artery stenosis and its risk factors.

## Conclusions

In conclusion, findings of the present study add to the previously published body of evidence suggesting that the serum chemerin and Hcy levels of ICVD patients were highly expressed, and were closely linked to the changes of the disease. TG, LDL-C, chemerin and Hcy were regarded as independent influencing factors of ICVD. Hcy and chemerin should be monitored in patients suspected of ICVD.

## Data Availability

The datasets generated and analyzed during the current study are available from the corresponding author on reasonable request.
